# Treatment Effects of Xuebijing Injection in Severe Septic Patients with Disseminated Intravascular Coagulation

**DOI:** 10.1155/2014/949254

**Published:** 2014-03-23

**Authors:** Qin Yin, Chunsheng Li

**Affiliations:** Emergency Department, Beijing Chaoyang Hospital, Capital Medical University, No. 8 Workers' Stadium South Road, Beijing 100020, China

## Abstract

The treatment effects of Xuebijing (XBJ) injection in severe septic patients with disseminated intravascular coagulation (DIC) were investigated in this study. 171 severe septic patients with DIC were divided into the control group (*n* = 83) or intervention group (*n* = 88). Routine therapies were administered in both groups, and XBJ injection was administered additionally in the intervention group. Incidence of DIC, clinical severity scores, and coagulation parameters at 7 days after administration of XBJ injection were compared between the two groups, and short-term prognosis was evaluated by 28-day mortality. Compared with the control group, incidence of DIC in the intervention group was significantly lower at 7 days after administration of XBJ injection (*P* < 0.001). In addition, differences of platelet count and prothrombin time were significantly greater in the intervention group than in the control group (*P* all <0.05), and similar results were also found for differences of the Mortality in Emergency Department Sepsis score and Acute Physiology and Chronic Health Evaluation II score (*P* all <0.05). Furthermore, 28-day mortality was significantly lower in the intervention group (*P* = 0.034). These results demonstrate that XBJ injection can effectively treat DIC caused by severe sepsis and improve short-term prognosis of severe septic patients with DIC.

## 1. Introduction

Despite advancements in modern antibiotics and supportive therapies, the mortality of severe sepsis remains high. In the United States, severe sepsis accounts for 751,000 hospital admissions and 215,000 deaths every year, with an in-hospital mortality of 28.6% [1]. Disseminated intravascular coagulation (DIC), a serious complication of severe sepsis, is characterized by widespread fibrin deposition in microvessels resulting from coagulation activation, inhibition of anticoagulation and fibrinolysis, and subsequent consumption of clotting factors and hyperfibrinolysis [2]. DIC is closely associated with the development of multiple organ failure, and mortality in patients with DIC is much higher than in those without DIC [3].

Current management of DIC relies on treating the underlying disease aggressively, along with supplementary therapy with clotting factors and platelets as required [4]. In addition, anticoagulation is also the main target of treatment. Heparin is the most widely used anticoagulant in clinical practice, but controlled trials did not confirm the survival benefit of heparin [5, 6]. Another anticoagulant, recombinant human activated protein C (rhAPC), was reported to decrease mortality in the Recombinant Human Activated Protein C Worldwide Evaluation in Severe Sepsis (PROWESS) study [7], and post hoc data analysis demonstrated that patients with DIC may have a survival benefit in particular [8]. However, this drug was found not to offer a survival benefit in the recent PROWESS-SHOCK study [9]. In the latest guidelines of Surviving Sepsis Campaign, rhAPC has been withdrawn [10]. Taken together, there is an urgent need for new effective therapies for DIC.

Xuebijing (XBJ) injection is an intravenous preparation made from five traditional Chinese medicines, namely, Chishao (Radix Paeoniae Rubra), Danggui (Radix Angelica Sinensis), Chuanxiong (Rhizoma Chuanxiong), Honghua (Flos Carthami), and Danshen (Radix Salviae Miltiorrhizae). The bioactive roles of XBJ injection include activating circulation, removing blood stasis, and clearing away toxins [11]. Previous studies have confirmed that XBJ injection is effective for treating sepsis [12], and this drug has been formally approved by the State Food and Drug Administration of China for use in clinical practice.

Although the effects of XBJ injection for treating sepsis have been established, there are few studies on XBJ injection in DIC. Some studies have shown that XBJ injection is also effective in treating patients with DIC [13, 14]. However, the dose and time of XBJ injection were not uniform, and the sample sizes were relatively small in these studies. Therefore, the main purpose of this study was to investigate the treatment effects of XBJ injection on DIC caused by severe sepsis.

## 2. Patients and Methods

### 2.1. Patients and Settings

This study included 171 adult severe septic patients with DIC who were admitted to the emergency intensive care unit (EICU) of Beijing Chaoyang Hospital, which is an urban, university hospital, from January 2011 to December 2011. Exclusion criteria were as follows: age <18 years, survival time <7 days after EICU admission, previous history of coagulopathy, use of anticoagulants, pregnancy, or breast feeding.

According to the criteria of the 2001 SCCM/ESICM/ACCP/ATS/SIS International Sepsis Definitions Conference [15], systemic inflammatory response syndrome (SIRS) was defined with two or more of the following criteria: (1) body temperature >38.3°C or <36°C; (2) heart rate >90 beats per minute; (3) respiratory rate >20 breaths per minute or PaCO_2_ <32 mmHg; (4) white cell count >12 × 10^9^/L or <4 × 10^9^/L or the percentage of immature neutrophil >10%. Sepsis was defined as SIRS caused by infection. Severe sepsis was defined as sepsis-induced acute organ dysfunction. The criteria of acute organ dysfunction were as follows: (1) sepsis-induced hypotension; (2) lactate above normal upper limits; (3) urine output <0.5 mL/kg/hour for more than 2 hours despite adequate fluid resuscitation or creatinine >2.0 mg/dL (176.8 *μ*mol/L); (4) acute lung injury with PaO_2_/FiO_2_ <250 mmHg in the absence of pneumonia as infection source or acute lung injury with PaO_2_/FiO_2_ <200 mmHg in the presence of pneumonia as infection source; (5) bilirubin >2.0 mg/dL (34.2 *μ*mol/L); and (6) platelet count <100,000 *μ*L or international normalized ratio (INR) >1.5.

DIC was diagnosed according to the criteria of the International Society on Thrombosis and Haemostasis Subcommittee. Platelet count, prothrombin time (PT), fibrinogen, and D-dimer levels were used to calculate the DIC score, and a score ≥5 was considered compatible with overt DIC (Table 1) [16].

### 2.2. Methods

#### 2.2.1. Study Design

As this was a retrospective study, ethical approval and informed consent were not necessary according to the Institutional Review Board of Beijing Chaoyang Hospital. The available database of laboratory parameters and the clinical database of our EICU were used in this study.

#### 2.2.2. Treatment Regiment

The mainstays of routine treatments for severe septic patients with DIC were antibiotics, fluid resuscitation, mechanical ventilation, vasoactive agents, nutritional support, and transfusion of blood products, whereas coagulation inhibitors and anticoagulants were not used in our EICU. Patients were divided into the control group or intervention group. For patients in the control group, only routine treatments were administered. For patients in the intervention group, besides routine treatments, 100 mL XBJ injection (Tianjin Chasesun Pharmaceutical Co., Ltd., Tianjin, China) with 100 mL normal saline was administered via intravenous drip twice daily for 7 consecutive days. XBJ injection was administered separately, and 50 mL normal saline was used to wash the pipeline before and after administration of XBJ injection. Therefore, XBJ injection did not interact with other routine medicines.

#### 2.2.3. Evaluation of Treatments

The primary efficacy endpoint was incidence of DIC as evaluated at 7 days after administration of XBJ injection. Other evaluation parameters were as follows: (1) clinical severity scores, including the Mortality in Emergency Department Sepsis (MEDS) score and Acute Physiology and Chronic Health Evaluation (APACHE) II score; (2) coagulation parameters, including platelet, PT, fibrinogen, D-dimer, and INR; and (3) differences of clinical severity scores and coagulation parameters (values at baseline minus values at 7 days after administration of XBJ injection).

Nine variables of the MEDS score were as follows: terminal illness, tachypnea or hypoxia, septic shock, low platelet count, bandemia, age >65 years, lower respiratory tract infection, nursing home resident, and altered mental status. The MEDS score was calculated by summing the points of nine variables, giving a possible score of 0 to 27 [17].

The APACHE II score comprised weightings for temperature, mean arterial pressure, heart rate, respiratory rate, oxygenation, arterial pH, sodium, potassium, creatinine, hematocrit, white blood cell count, Glasgow coma score, age, chronic diseases, and surgical status, giving a possible score of 0 to 71 [18].

PT, INR, fibrinogen, and D-dimer levels were measured on sodium citrate anticoagulated samples using Sysmex CA7000 blood coagulation analyzer (Sysmex Corporation, Kobe, Japan). PT, INR, and plasma fibrinogen levels were measured with coagulation-based activity assay. Plasma D-dimer levels were measured with the method of immunoturbidimetric assay. Platelet count was measured on EDTA anticoagulated samples with the method of fluorescent dye laser scattering using XE-2100 blood analyzer (Sysmex Corporation, Kobe, Japan).

#### 2.2.4. Evaluation of Side Effects of XBJ Injection

Evaluation parameters of side effects of XBJ injection included (1) changes in vital signs; (2) complete blood cell count and urine routine examination; and (3) biochemical parameters, including hepatic function (aspartate aminotransferase, alanine aminotransferase, total bilirubin, and direct bilirubin), renal function (blood urea nitrogen and creatinine), glucose, and electrolytes.

#### 2.2.5. Outcome of the Study

The primary outcome of this study was all-cause 28-day mortality, and the survival or death of patients within 28 days was recorded.

### 2.3. Statistical Analysis

The results were analyzed with SPSS 16.0 software (SPSS Inc., Chicago, IL, USA). Normally distributed data were expressed as the mean ± standard deviation and nonnormally distributed data were expressed as the median (25–75th percentiles). The Student paired *t*-test was used to compare normally distributed data between two groups, and the rank sum test was applied for nonnormally distributed data between two groups. The *χ* ^2^ test was used to compare frequencies. All reported *P* values were two-sided, and *P* < 0.05 was considered statistically significant.

## 3. Results

### 3.1. Baseline Characteristics of Patients

This study included 171 severe septic patients with DIC: 83 patients in the control group and 88 patients in the intervention group. The baseline characteristics of the overall study population are shown in Table 2. Differences of demographical data between the two groups were not statistically significant (*P* all >0.05) (Table 2). In addition, the MEDS score and APACHE II score at baseline did not differ significantly between the two groups, and similar results were also found for coagulation parameters at baseline (*P* all >0.05) (Table 2).

### 3.2. Incidence of DIC at 7 Days after Administration of XBJ Injection

Compared with the control group, incidence of DIC in the intervention group was significantly lower at 7 days after administration of XBJ injection (*P* < 0.001) (Table 3).

### 3.3. Clinical Severity Scores and Coagulation Parameters at 7 Days after Administration of XBJ Injection

Compared with the control group, the MEDS score was lower in the intervention group (*P* = 0.039), and the APACHE II score did not differ significantly between the two groups at 7 days after administration of XBJ injection (*P* = 0.107) (Table 3). In addition, PT was shorter in the intervention group (*P* = 0.003), and another four coagulation parameters did not differ significantly between the two groups at 7 days after administration of XBJ injection (*P* all >0.05) (Table 3).

### 3.4. Differences of Clinical Severity Scores and Coagulation Parameters (Values at Baseline Minus Values at 7 Days after Administration of XBJ Injection)

Differences of the MEDS score and APACHE II score were greater in the intervention group than in the control group (*P* all <0.05) (Table 4). In addition, differences of platelet count and PT were greater in the intervention group (*P* all <0.05), and differences of another three coagulation parameters were not significant between two groups (*P* all >0.05) (Table 4).

### 3.5. 28-Day Mortality

Compared with the control group, 28-day mortality was significantly lower in the intervention group (*P* = 0.034) (Table 2).

### 3.6. Side Effects of XBJ Injection

There were no records of side effects during administration of XBJ injection.

## 4. Discussion

The main findings of this study were as follows: (1) compared with the control group, incidence of DIC was significantly lower in the intervention group at 7 days after administration of XBJ injection; and (2) 28-day mortality was significantly lower in the intervention group than in the control group.

XBJ injection is a compound preparation made from five traditional Chinese medicines. Consistency in the quality of XBJ injection among different batches is ensured by fingerprint technology, which refers to the use of spectroscopy and chromatography to obtain the characteristics of component groups, maps, or images, combined with computer technology to analyze information, thereby identifying the authenticity of the drugs [19]. Multiple bioactive constituents, such as safflower yellow A, ferulic acid, and Danshensu, are identified in XBJ injection, and these constituents are responsible for the therapeutic effects of XBJ injection [20, 21].

Currently, there are few studies on XBJ injection for treating DIC. Furthermore, the dose and time of XBJ injection in previous studies were not uniform. The report of Guo et al. included 4 patients with DIC, among whom two received 100 mL XBJ injection twice daily, one received 100 mL XBJ injection once daily, and one received 200 mL XBJ injection twice daily [13]. In Chen's study, 30 patients with DIC received 100 mL XBJ injection twice daily, but the administration time ranged from 7 to 14 days [14]. According to the instructions of this drug, 100 mL XBJ injection with 100 mL normal saline twice daily for 7 consecutive days is recommended for patients with organ dysfunction caused by infection; on the other hand, three or four times daily administration is allowed, and administration time may be ranged from 7 to 14 days for very critically ill patients. On the basis of these recommendations, the routine dose and time of XBJ injection for patients with severe sepsis are 100 mL XBJ injection with 100 mL normal saline twice daily for 7 consecutive days in our EICU. Therefore, this dose and time were selected in this study.

It has been recognized that the crosstalk between inflammation and coagulation is important in the pathogenesis of sepsis, and endothelium is the central link. During sepsis, inflammatory mediators cause endothelial damage, and damaged endothelium manifests enhanced procoagulatory properties and leads to coagulation activation; vice versa coagulation activation enhances inflammatory responses. DIC is the most severe form of coagulation abnormalities and its development is closely associated with endothelial damage [2]. Previous clinical studies have confirmed that XBJ injection can alleviate the extent of endothelial damage in critically ill patients [22, 23]. In addition, XBJ injection can inhibit coagulation activation [24, 25] and reduce inflammatory responses [26, 27]. In this study, we found that, compared with the control group, incidence of DIC was significantly lower in the intervention group at 7 days after administration of XBJ injection (Table 3). Furthermore, differences of platelet count and PT between values at baseline and 7 days after administration of XBJ injection were significantly greater in the intervention group (Table 4). Taken together, the therapeutic effects of XBJ injection on DIC may be attributed to its protective roles on the endothelium and are mainly dependent on increasing platelet count and decreasing PT.

The MEDS score and APACHE II score at baseline were not significantly different between the two groups (Table 2), indicating that the disease severity of patients in the two groups was comparable before treatment. However, after treatment, the MEDS score was significantly lower in the intervention group than in the control group (Table 3). Furthermore, differences of the MEDS score and APACHE II score between values at baseline and 7 days after administration of XBJ injection were significantly greater in the intervention group (Table 4). These results demonstrate that XBJ injection may obviously alleviate the disease severity in severe septic patients with DIC. Previous studies have shown that incidence of DIC increases with the severity of sepsis [28, 29]. Therefore, these results may be associated with the effects of XBJ injection on DIC.

28-day mortality in the intervention group and control group was 20.45% and 34.94%, respectively. It is notable that 28-day mortality was significantly lower in the intervention group (Table 2). Previous studies have demonstrated that mortality in septic patients with DIC is significantly higher than in those without DIC, and resolution of DIC is associated with a favorable outcome [30, 31]. In the present study, incidence of DIC was significantly lower after administration of XBJ injection, confirming that this drug is effective in improving short-term prognosis of severe septic patients with DIC.

An important limitation of this study is the retrospective design. Although the data were analyzed on the basis of laboratory parameters at entry and after treatments, this type of analysis should always be interpreted with care. Another important limitation is that this was a single-center study, and the results may not be applicable to other hospitals. Therefore, a prospective, multicenter, randomized controlled trial is needed to further confirm the effects of XBJ injection in severe septic patients with DIC in future research.

## 5. Conclusions

In conclusion, this study demonstrates that XBJ injection can effectively treat DIC caused by severe sepsis and improve short-term prognosis of severe septic patients with DIC.

## Figures and Tables

**Table 1:** ISTH scoring system for overt DIC.

	0	1 point	2 points	3 points
Platelet (×10^9^/L)	≥100	<100 but ≥50	<50	
Prolongation of PT	≤3 sec	>3 sec but ≤6 sec	>6 sec	
Fibrinogen (g/L)	≥1.0	<1.0		
D-dimer (mg/L)	≤0.4		>0.4 but ≤4	>4

ISTH: International Society on Thrombosis and Haemostasis; DIC: disseminated intravascular coagulation; PT: prothrombin time.

**Table 2:** Baseline characteristics of patients.

	Control group (*n* = 83)	Intervention group (*n* = 88)	*P*
Age (yrs)	56 (49–73)	59 (53–74)	0.14
Male (*n*, %)	51 (61.45)	57 (64.77)	0.652
BMI	23.67 ± 2.93	22.97 ± 3.40	0.158
28-day mortality (*n*, %)	29 (34.94)	18 (20.45)	0.034
Platelet (×10^9^/L)	79.61 ± 49.69	70.51 ± 58.92	0.326
PT (s)	25.72 ± 7.34	22.66 ± 8.46	0.263
Fibrinogen (g/L)	2.64 ± 1.47	2.57 ± 1.93	0.17
D-dimer (mg/L)	4.7 (1.85–8.62)	5.2 (2.12–9.53)	0.192
INR	1.79 ± 0.91	1.71 ± 0.88	0.607
MEDS score	11 (8–19)	12 (9–21)	0.794
APACHE II score	24 (15–32)	26 (16–35)	0.334
Type of infection (*n*, %)			
Pneumonia	39 (46.88)	49 (55.68)	0.256
IAI	37 (44.58)	33 (37.5)	0.347
USI	7 (8.43)	6 (6.82)	0.69

BMI: body mass index; PT: prothrombin time; INR: international normalized ratio; MEDS score: Mortality in Emergency Department Sepsis score; APACHE II score: Acute Physiology and Chronic Health Evaluation II score; IAI: intra-abdominal infection; USI: urinary system infection.

**Table 3:** Incidence of DIC, clinical severity scores, and coagulation parameters at 7 days after administration of Xuebijing injection.

	Control group (*n* = 83)	Intervention group (*n* = 88)	*P*
DIC (*n*, %)	22 (26.51)	5 (5.68)	<0.001
Platelet (×10^9^/L)	119.15 ± 91.24	131.95 ± 88.36	0.205
PT (s)	21.52 ± 14.81	17.2 ± 8	0.003
Fibrinogen (g/L)	3.65 ± 1.63	3.67 ± 1.56	0.183
D-dimer (mg/L)	3.72 (1.26–6.37)	3.45 (1.12–5.87)	0.743
INR	1.65 ± 0.83	1.65 ± 0.93	0.997
MEDS score	8 (5–18)	7 (4–15)	0.039
APACHE II score	14 (11–27)	13 (10–25)	0.107

DIC: disseminated intravascular coagulation; MEDS score: Mortality in Emergency Department Sepsis score; APACHE II score: Acute Physiology and Chronic Health Evaluation II score; PT: prothrombin time; INR: international normalized ratio.

**Table 4:** Differences of clinical severity scores and coagulation parameters (values at baseline minus values at 7 days after administration of Xuebijing injection).

	Control group (*n* = 83)	Intervention group (*n* = 88)	*P*
Platelet (×10^9^/L)	−38.95 ± 76.06	−61.44 ± 81.84	0.005
PT (s)	4.25 ± 6.32	5.46 ± 7.36	0.036
Fibrinogen (g/L)	−0.99 ± 4.38	−1.11 ± 4.08	0.563
D-dimer (mg/L)	1.24 ± 2.02	1.63 ± 2.21	0.138
INR	0.16 ± 0.65	0.08 ± 0.73	0.472
MEDS score	2.05 ± 3.98	3.85 ± 4.63	0.007
APACHE II score	7.18 ± 8.97	10.63 ± 9.17	0.014

MEDS score: Mortality in Emergency Department Sepsis score; APACHE II score: Acute Physiology and Chronic Health Evaluation II score; PT: prothrombin time; INR: international normalized ratio.
